# Distinct Functional Network Connectivity for Abstract and Concrete Mental Imagery

**DOI:** 10.3389/fnhum.2018.00515

**Published:** 2018-12-18

**Authors:** Sobhan Hemati, Gholam-Ali Hossein-Zadeh

**Affiliations:** ^1^School of Electrical and Computer Engineering, College of Engineering, University of Tehran, Tehran, Iran; ^2^School of Cognitive Sciences, Institute for Researches in Fundamental Sciences (IPM), Tehran, Iran

**Keywords:** concrete words, abstract words, language, functional network connectivity, group ICA, fMRI

## Abstract

In several behavioral psycholinguistic studies, it has been shown that concrete words are processed more efficiently. They can be remembered faster, recognized better, and can be learned easier than abstract words. This fact is called concreteness effect. There are fMRI studies which compared the neural representations of concrete and abstract concepts in terms of activated regions. In the present study, a comparison has been made between the condition-specific connectivity of functional networks (obtained by group ICA) during imagery of abstract and concrete words. The obtained results revealed that the functional network connectivity between three pairs of networks during concrete imagery is significantly different from that of abstract imagery (FDR correction at the significance level of 0.05). These results suggest that abstract and concrete concepts have different representations in terms of functional network connectivity pattern. Remarkably, in all of these network pairs, the connectivity during concrete imagery is significantly higher than that of abstract imagery. These more coherent networks include both linguistic and visual regions with a higher engagement of the right hemisphere, so the results are in line with dual coding theory. Additionally, these three pairs of networks include the contrasting regions which have shown stronger activation either in concrete or abstract word processing in former studies. The findings imply that the brain is more integrated and synchronized at the time of concrete imagery and it may explain the reason of faster concrete words processing. In order to validate the results, we used functional network connectivity distributions (FNCD). Wilcoxon rank-sum test was used to check if the abstract and concrete FNCDs extracted from whole subjects are the same. The result revealed that the corresponding distributions are different which indicates two different patterns of connectivity for abstract and concrete word processing. Also, the mean of FNCD is significantly higher at the time of concrete imagery than that of abstract imagery. Furthermore, FNCDs at the single-subject level are significantly more left-skewed or equally, include more strong connectivity for concrete imagery.

## Introduction

Concrete features include features of an object/creature which are more imageable and give information about the physical properties, e.g., the neck of an animal. In contrast, abstract features are less imageable which are related to conceptual features of an object or animal, e.g., kind or cruel animal.

There is some evidence which implies that processing of concrete features is faster, more precise, and less vulnerable to brain damage in compare with the processing of abstract features (Paivio, 1991; Schwanenflugel, 1991). The fact that people are able to remember, learn, and recognize concrete words easier than abstract words, called concreteness effect. This effect is more significant in patients with aphasia caused by left-hemispheric damage (Goodglass et al., 1969) and occurs in different mental imagery tasks as well (Jessen et al., 2000). Two models have been introduced to explain concreteness effect; “context availability” and “dual coding theory.” The context availability theory argues that the superior processing of concrete words compared to abstract words is caused by the easier recall of concrete contexts (Schwanenflugel et al., 1992). The dual coding theory claims that the processing of abstract words is merely based on verbal code representations of the left cerebral hemisphere whereas, concrete nouns have access to both left cerebral hemisphere as well as image-based processing system in the right hemisphere (Paivio, 1991). In this point of view, abstract concepts are related to only linguistic areas of the brain whereas both linguistic and visual regions have significant roles in the processing of concrete words. There are two lines of evidence about dual coding theory; first, studies which support this idea that right hemisphere is much more involved in the processing of concrete words rather than abstract words (Day, 1979). Second, studies based on physiological (Howell and Bryden, 1987; Martín-Loeches et al., 2001), and neuroimaging (Fiebach and Friederici, 2004) experiments provided evidence against the first theory. It would be invaluable to compare representations of concrete and abstract concepts in the brain (Papagno et al., 2013). Understanding the mechanism of processing the concrete and abstract words in the brain can increase our insight about language function in both healthy and patient subjects (Eviatar et al., 1990; Kuperberg et al., 2008).

Functional magnetic resonance imaging (fMRI) has been used for many years to study the brain through blood oxygen level dependent (BOLD) changes during various cognitive tasks. fMRI has been used in some studies to extract activated regions during the processing of concrete and abstract concepts and determine the differences between them. Previous studies reported different spatial localizations of abstract and concrete words processing (Wang et al., 2010). For example, abstract words produced higher activation, particularly in the left superior temporal and inferior frontal cortex of the brain. The brain areas which had greater activation during concrete word processing are located in a bilateral network of associative areas, including parietal, temporal and prefrontal cortex, both during a semantic similarity (Sabsevitz et al., 2005) and synonym judgment task (Noppeney and Price, 2004). In one fMRI study, D'Esposito et al. (1997) showed mental imagery of concrete words lead to higher activation in some areas in left hemisphere including the fusiform gyrus, premotor area, and anterior cingulate gyrus compared to abstract word imagery whereas superior frontal gyrus and precuneus both in right hemisphere had a stronger activation during imagery of abstract words. In a lexical decision paradigm, Binder et al. (2005) found areas in the left lateral temporal lobe which were activated equally by both abstract and concrete types of auditory presented words. Bilateral regions including the angular gyrus and dorsal prefrontal cortex are more involved in processing concrete words, whereas left inferior frontal regions, which are related to phonological and verbal working memory processes, had a higher amplitude of activation by abstract words. There are studies which only identified areas with greater activation for abstract words compared to concrete concepts (Kiehl et al., 1999; Perani et al., 1999; Friederici et al., 2000). These studies failed to provide evidence for the right hemisphere engagement during processing of concrete words that is predicted by dual code theory.

In short, according to former studies, the brain areas which consistently showed stronger activation at the time of concrete word processing compared to abstract are superior occipital gyrus, middle occipital gyrus, fusiform gyrus, posterior cingulate, and angular gyrus (Whatmough et al., 2004; Sabsevitz et al., 2005; Wang et al., 2010). Abstract concepts led to stronger activity in superior temporal gyrus, middle temporal gyrus, and inferior frontal gyrus (Mellet et al., 1998; Wang et al., 2010). There are inconsistent results about precuneus and precentral gyrus. Many studies found stronger activation for precuneus during concrete word processing (Jessen et al., 2000; Binder et al., 2005). However, in a study by D'Esposito et al. (1997) this region showed stronger activation at the time of abstract word processing. Although abstract word processing has led to stronger activation in precentral gyrus in some studies (Binder et al., 2005; Wang et al., 2010), in a study by Mellet et al. (1998) higher activation reported for this region during concrete word processing. These inconsistent results could be originated from different task paradigms or experiment methodologies.

Another approach based on multi-voxel pattern analysis (MVPA) (Norman et al., 2006), has been utilized in order to peruse mental imagery in the context of concrete and abstract word processing. Recently, MVPA technique has been applied to a semantic similarity judgment task in order to decode fMRI data and identify whether the individual trials are abstract or concrete (Wang et al., 2013). In that study, authors showed that the classification accuracy for abstract and concrete trials was significantly above the chance level. These results suggest that the spatial patterns of brain activation during abstract and concrete word processing are different. As far as the authors of this paper are concerned less research have tried to study the brain functional connectivity in abstract and concrete word processing. Recently, a group graphical model has been used to study brain functional connectivity between eight pre-defined brain regions in a lexical decision task (Westbury et al., 2016). In this study, high imageability/concreteness showed additional connections between the left and right angular gyri and between the left angular gyrus and the left cingulate. One extra connection for the low imageability/concreteness condition was found and included the inferior frontal gyrus-superior temporal gyrus connection. Recently, generalized psychophysiological interaction analyses (Della Rosa et al., 2018) revealed the functional correlations of the intrinsic and extrinsic properties (imageability and context availability) of abstract and concrete representations with brain activation differentiating abstract and concrete representations. In this study, the connectivity strength values extracted from each region connected with the left inferior frontal gyrus were correlated with the activity of this area for abstract words, and a regression analysis was conducted to highlight which areas recruited by low imageability or low context availability predicted the greater activation of the inferior frontal gyrus for abstract concepts. Only the left middle temporal gyrus/angular gyrus, which are engaged in semantic processing, was a significant predictor of left inferior frontal gyrus activity and differentiated abstract from concrete words.

According to dual-coding theory, more brain areas are involved in the process of concrete words in compare with abstract words. These include left cerebral hemisphere, as well as image-based processing system in the right hemisphere. To the best of our knowledge, fewer works have studied abstract/concrete word processing from a functional connectivity point of view. In this paper, we try to examine whether higher engagement of the brain (including both hemispheres) during concrete imagery affects functional connectivity among brain networks. To this aim, a non-parametric multivariate method based on group spatial Independent Component Analysis is utilized (ICA) (McKeown et al., 1998; Calhoun et al., 2001). In this method, no anatomical information is used to extract brain networks (group spatial independent components) during the performance of the abstract-concrete mental imagery task. fMRI data was acquired from a group of healthy subjects while they were evaluating the imagined animal characteristics. The first block is used to asses abstract and the second one is used for evaluating concrete animals' characteristics. The spatial independent components (i.e., networks) which are extracted using group spatial ICA, are spatially independent but their corresponding time-courses have a substantial temporal dependency. The amount of synchronization between different networks may be used to evaluate the functional connectivity. In the present research, there has been an effort to answer the key question “whether the functional network connectivity (FNC) (Jafri et al., 2008) during imagery of abstract characteristics is different from that of concrete imagery.” If so, between which pairs of the networks, the connectivity is significantly different.

## Material and Method

### Participants

14 healthy volunteers with no history of surgery, neurological or psychiatric disorder participated in the study (nine men and five women age 26.7 **±** 6). The study was approved by an institutional review board (IRB) of the Institute for Research in Fundamental Sciences (IPM). As requested by ethical committee all participants signed a written consent form prior to the beginning of the experiment session. The participants were instructed to close their eyes in the MRI scanner while they performed visual mental imagery guided by auditory commands given to them (in the Persian language) through an MR compatible headphone.

### Stimuli

**Figure 1:**
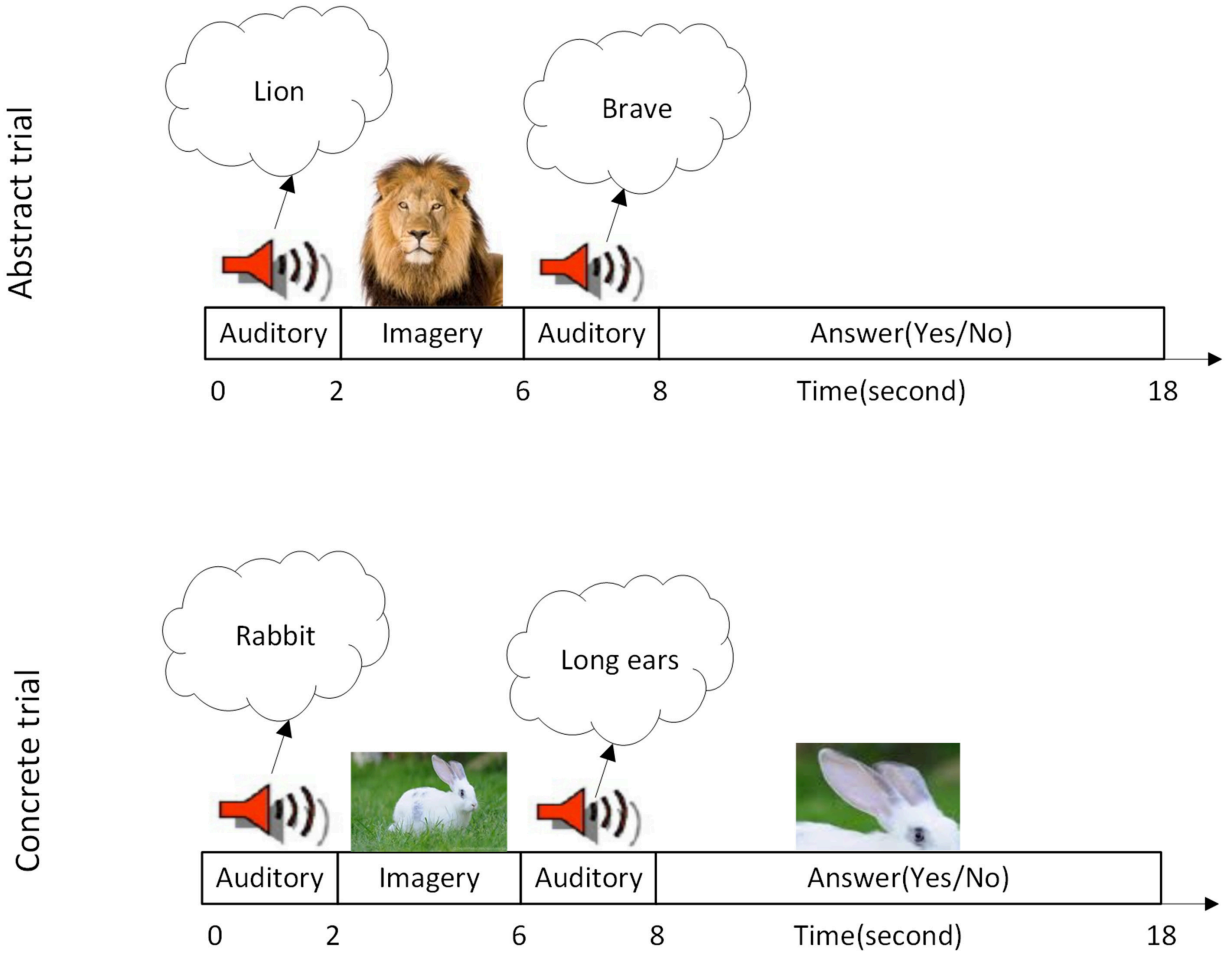
Task specification. **(Top Row)** A single imagery trial of the abstract characteristic. **(Bottom Row)** A single imagery trial of the concrete characteristic is illustrated. At the time of each trial, subjects were told first to imagine a named animal. Then they were asked to evaluate whether the named animal has the mentioned characteristic or not. Each block contains 12 trials so each block of task lasting 216 s.

The fMRI paradigm has two main blocks, and it is similar to the task which has been used in former studies (Klein et al., 2000; Goldberg et al., 2007). The first block includes 12 trials of mental imagery of abstract characteristics (with low concreteness and imageability) and the second one have 12 trials of imagery of concrete characteristics (with high concreteness and imageability). In the current paper, imageability and concreteness are considered as the same measure, because these two correlate to a high degree (specifically in the dataset which has been used for this study). To control the semantic content of every verification decision, all items and properties were drawn from the category of animals. A total of 60 (Persian) animal names which were frequently used and had between one and three syllables were selected. One abstract and one concrete characteristic (in Persian) were considered for each animal. Abstract characteristics in the first block were related to semantic features of the animals (such as “brave”) and concrete characteristics in the second block were related to outward details of animals (like “long ears”). Twenty students were asked to rate the concreteness of the animal characteristic depending on whether the characteristic relies more on directly perceived experiences and it is more imageable or indirectly learned verbal knowledge, and it is less imageable (see Tables S1, S2 of Supplementary Material). The scales ranged from 1 to 7, in which 1 indicated highly abstract (difficult to image) and 7 indicated highly concrete (easy to image). Also an abstract characteristic is defined for the participants as one that would be typically learned indirectly through verbal facts and cannot be experienced through their sense or action, whereas a concrete characteristic was explained as one that could be directly perceived through one of the five senses. The mean of concreteness for concrete words (mean, 6.0469 and *SD* 1.3014) was significantly higher (*p*≅0) in compare with abstract words (mean, 2.8125 and *SD* 1.7427). Each animal name appeared equally often with each type of characteristics and appeared only once with each type of characteristics. Both animals and characteristics were presented in a random order. Figure 1 shows one trial of abstract and one trial of concrete characteristics mental imagery in this paradigm, respectively. In each trial, the name of an animal was presented to the subject (through MR compatible headphone). Then she/he had 4 s to establish a visual mental image of that animal. In the next step, the subject heard a possible characteristic of the animal in 2 s. Then the subject had 10 s to decide, whether or not the animal has that characteristic (in 50% of presented cases animals had the characteristics) and press “yes” or “no” key of a response box. Thus, the duration of each trial is 18 s.

### FMRI Data Acquisition

Both structural and functional images were collected using a Siemens Trio Tim 3 T MRI scanner located in Imam Hospital in Tehran. T1-weighted anatomical images were collected for anatomical registration of the functional data. These images were acquired using a high resolution three-dimensional T1-weighted MPRAGE pulse sequence (TR/TE = 1, 800/3.44 *ms*, flip angle = 7, voxel size of 1 × 1 × 1 *mm*^3^, and a matrix size *of* 256 × 256 × 190). Functional MR images were acquired using echo planar imaging (EPI) sequence (TR/TE = 2, 000/30 *ms*, flip angle = 90°, voxel size 3 × 3 × 4 *mm*^3^, matrix size 64 × 64, and 30 slices per volume, 216 volume image).

### Preprocessing

In the first step, the first six EPI volume images were removed. Also, the data of the two subjects were omitted from further analysis because some parts of the parietal lobe of their brains were out of the imaging field of view. Data pre-processing was performed using FEAT fMRI analysis tool in FSL (FMRIB's Software Library, www.fmrib.ox.ac.uk/fsl) toolbox (Smith et al., 2004; Woolrich et al., 2009; Jenkinson et al., 2012) and consist of: interleaved slice timing correction, motion correction using MCFLIRT (Jenkinson et al., 2002), brain extraction using the Brain Extraction Tool (BET) (Smith, 2002), temporal high-pass filtering (120 s) and spatially smoothed with a 8^*^8^*^8 *mm*^3^ the full width of the half-maximum Gaussian kernel. EPI data of each subject was firstly registered to corresponding T1-weighted anatomical images and then were normalized into the MNI space using FLIRT (FMRIB's linear image registration tool) (Jenkinson and Smith, 2001).

### Group ICA

In this research, ICA is utilized as an exploratory method to study the whole brain, and no prior anatomical information is used in extracting functional networks of the brain and their synchronization during imagery of abstract and concrete characteristics. To apply group ICA, after the preprocessing stage, the functional data of every subject were registered to MNI space and we were able to perform group ICA. Note that the terms “networks,” “components,” and “ICs” refer to the same concepts in this manuscript. The functional networks were estimated by group spatial ICA (Calhoun et al., 2001) using GIFT software (http://icatb.sourceforge.net/). As a result, the group spatial ICs were extracted from all subjects. Corresponding to every specific IC, each subject's time courses were extracted using back reconstruction technique (Calhoun et al., 2001). Before applying ICA, the data dimension of each subject was reduced using PCA (to 53 principal components) then all single subject's data were concatenated temporally, and the dimension of this aggregated data was reduced. The number of independent components (components in aggregated data), was estimated by modified minimum description length criteria (Li et al., 2007), and was equal to 35 ICs. Then ICA is performed by minimizing the mutual information between spatial components using the Infomax algorithm (Bell and Sejnowski, 1995).

### Component Inspection

Spatial group ICA derived 35 group independent networks from data. These networks can be classified into two categories which are functional networks during the task and the artifacts or noise representative networks. In the first stage, the networks corresponding to noise, eye movements and head motion artifacts were identified (components number 1, 5, 9, 11, 12, 14, 15, 16, 19, 29, 34, 35) using visual inspection of all 35 ICs and excluded from further analysis.

### Functional Network Connectivity

Each independent component is identified by a spatial map and a temporal loading, which represents the temporal fluctuations of that IC over time, Each IC is usually considered as a functional network. Different networks (corresponding to ICs) are spatially independent, but their temporal loadings may have a temporal correlation which is defined as functional network connectivity (FNC). As a result, the amount of FNC which has been proposed by Jafri et al. (2008) could be a good measure to study brain integration. The data contains 210 time points (after omitting the first 6 volume images) and has two main blocks for every subject. The first block involves 102 time points (12 abstract trials) and the second one contains 108 points (12 concrete trials). Easily, independent components time courses also contain 210 time points. For further analysis, it is better to consider the same number of time points for both blocks. To this aim, the first 6 time points of the second block in all network's time courses were omitted. Then, for every subject and block, a 23 by 23 connectivity matrix was built which measured the pair-wise correlation between corresponding ICs. This procedure was done for all subjects, so that for each state 12 connectivity matrices (corresponding to 12 subjects) were made. After obtaining these matrices, the connectivity of every network pair was compared during imagery of abstract and concrete characteristics. In other words for each connection (between a pair of networks), a 12 by 1 vector containing the connectivity in the concrete imagery was calculated (for all subjects) and another similar vector for imagery of abstract characteristics. The two-sided paired *t*-test was performed on these pairs of 12 by 1 vectors. There were 23 × (23−1)/2 = 253 connectivities to be tested for significance, so the FDR-adjustment (Genovese et al., 2002) at the *q* = 0.05 threshold has been used to avoid multiple testing errors.

## Results

**Figure 2:**
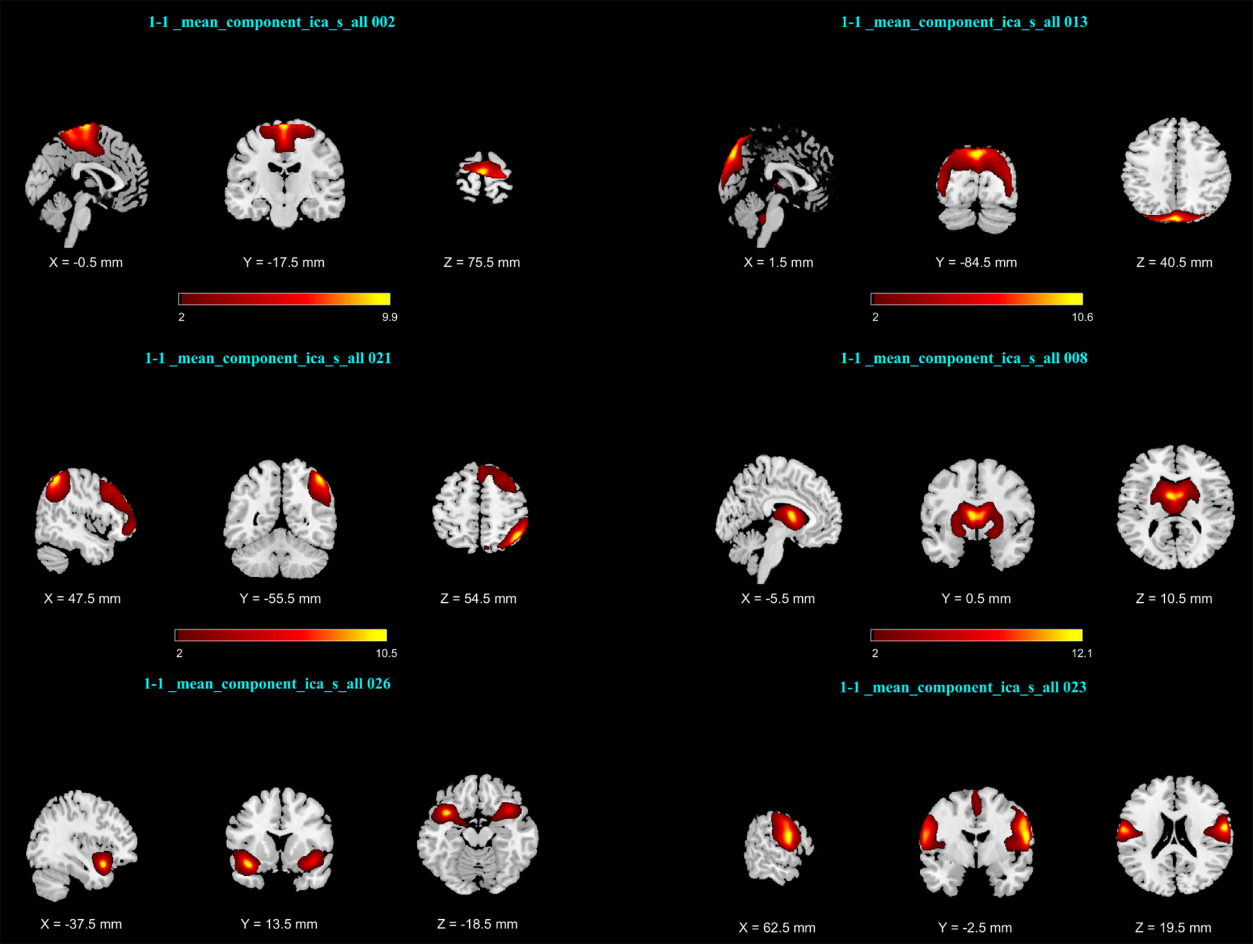
Activation maps of network pairs showing different connectivity between the mental imagery of concrete and abstract characteristics: **(Top Row)** IC number 2 and13; **(Middle Row)** ICs number 21 and 8; **(Bottom Row)** ICs number 26 and 23. Components were converted to z-scores and thresholded at *Z* > 2.

After calculating an FNC matrix for every subject and for each block of abstract or concrete characteristics, the connectivity of all possible pairs of networks was compared between two conditions using a two-sided paired *t*-test. This procedure revealed that the connectivity of three pairs (among 253 possible pairs) during the imagery of abstract characteristics was significantly different from that of concrete characteristics. These networks included ICs number 13 and 2 with *p* = 2.581 × 10^−4^; ICs number 21 and 8 with *p* = 1.974 × 10^−4^; and the ICs number 23 and 26 with *p* = 4.604 × 10^−6^ Figure 2 illustrates the spatial maps of these ICs and Table 1 shows the mentioned components which have been z-scored, along with the name of regions in the networks as well as the Brodmann areas (BA) survived after *Z*>2 threshold. As mentioned before, every component is a functional network. In other words, anatomic regions which have fallen in the specific component are functionally connected. Notably, the connection between all of these three pairs of networks during the mental imagery of concrete characteristics is higher than that of abstract characteristics. This shows that these networks works more coherent during imagery of concrete characteristics than abstract imagery.

**Table 1:** Brain regions of networks number 2, 13, 21, 8, 26, and 23 during abstract-concrete mental imagery.

**Region**	**Brodmann area**	**Volume (cc) L/R**	**Max z (x,y,z) L/R**	**Region**	**Brodmann area**	**Volume (cc) L/R**	**Max z (x,y,z) L/R**
**COMPONENT 2**				**COMPONENT 13**			
Paracentral lobule	4, 5, 6, 7, 31	6.3/5.1	7.2 (0, −36, 59)/7.7 (4, −37, 66)	Precuneus	7, 19, 31	3.7/4.7	8.4 (−8, −84, 39)/8.8 (4, −75, 46)
Medial frontal gyrus	6, 32	4.7/5.4	7.6 (−4, −14, 67)/7.5 (4, −14, 67)	Cuneus	7, 17, 18, 19	9.2/8.9	8.3 (−2, −80, 37)/7.6 (2, −78, 37)
Postcentral gyrus	1, 2, 3, 4, 5, 7, 40	3.6/4.0	6.5 (−6, −41, 65)/7.1 (6, −41, 65)	Middle occipital gyrus	18, 19, 37	7.2/6.8	7.7 (−20, −94, 21)/6.1 (32, −86, 21)
Precentral gyrus	4, 6	4.2/5.4	6.0 (−22, −20, 67)/5.8 (24, −22, 66)	Superior occipital gyrus	19	1.1/0.9	6.2 (−32, −86, 28)/6.0 (32, −86, 25)
Superior frontal gyrus	6	1.6/1.3	5.9 (−2, −6, 67)/5.3 (8, −16, 67)	Middle temporal gyrus	19, 21, 37, 39	1.7/2.7	4.7 (−44, −81, 21)/5.2 (40, −81, 21)
Sub-gyral	31, 40	2.2/2.8	4.7 (−12, −40, 61)/5.0 (12, −34, 62)	Inferior occipital gyrus	18, 19	0.9/0.7	3.7 (−40, −86, −1)/3.4 (40, −84, −3)
Cingulate gyrus	24, 31, 32	1.5/1.9	4.3 (0, −7, 45)/3.7 (4, −6, 44)	Lingual gyrus	18	0.3/0.1	3.4 (−26, −95, −4)/2.9 (8, −95, 1)
Superior parietal lobule	5, 7	0.3/0.1	3.8 (−20, −40, 61)/3.0 (24, −42, 61)	Inferior temporal gyrus	18, 19, 37	0.3/0.3	3.4 (−48, −76, −1)/3.2 (51, −70, 0)
Middle frontal gyrus	6	0.4/0.5	3.2 (−18, −14, 62)/3.6 (24, −14, 62)	Angular gyrus	39	0.2/0.3	2.9 (−44, −76, 31)/3.0 (44, −74, 33)
				Fusiform gyrus	19	0.1/0.4	2.5 (−44, −76, −11)/2.8 (46, −74, −11)
				Inferior parietal lobule	39	0.1/0.2	2.0 (−48, −66, 42)/2.6 (50, −66, 40)
				Sub-gyral		0.0/0.3	NS/3.2 (32, −78, 24)
**COMPONENT 21**				**COMPONENT 8**			
Inferior parietal lobule	7, 39, 40	0.0/10.9	NS/10.1 (48, −52, 52)	Extra-Nuclear		7.8/9.8	11.7 (−6, 2, 9)/9.5 (6, 0, 7)
Superior parietal lobule	7	0.0/1.8	NS/8.7 (44, −60, 51)	Lateral ventricle		3.3/3.2	11.0 (−4, 2, 5)/10.0 (2, 2, 7)
Postcentral gyrus	2, 40	0.0/0.6	NS/5.6 (55, −36, 52)	Caudate		4.0/4.1	10.8 (−8, 1, 13)/10.5 (6, 4, 7)
Supramarginal Gyrus	40	0.0/4.3	NS/5.5 (55, −49, 37)	Thalamus		2.8/2.6	9.0 (−6, −3, 13)/6.4 (4, −5, 9)
Superior frontal gyrus	6, 8, 9, 10, 11	0.0/10.3	NS/5.4 (32, 64, −3)	Lentiform nucleus		5.4/4.3	6.8 (−22, 10, −4)/6.3 (22, 9, −6)
Middle frontal gyrus	6, 8, 9, 10, 11, 46, 47	0.0/25.3	NS/5.4 (40, 59, 10)	Subcallosal gyrus	13, 34	0.3/0.4	3.8 (−20, 9, −11)/3.9 (22, 7, −12)
Angular gyrus	39	0.0/1.5	NS/5.0 (48, −58, 36)	Third ventricle		0.0/0.3	NS /3.5 (0, −6, 0)
Inferior frontal gyrus	9, 10, 45, 46	0.0/1.4	NS/4.6 (42, 58, 1)	Anterior cingulate	25	0.1/0.1	2.5 (−2, 6, −5)/3.2 (2, 10, −4)
Precentral gyrus	9	0.0/0.7	NS/4.4 (44, 25, 37)	Claustrum		0.1/0.1	2.6 (−28, 11, −6)/3.1 (30, 10, −4)
Medial frontal gyrus	6, 8, 9, 10	0.0/2.2	NS/3.6 (4, 35, 41)	Parahippocampal gyrus	34	0.1/0.3	2.1 (−20, 1, −14)/3.1 (20, 1, −14)
Superior temporal gyrus	39	0.0/0.3	NS/3.1 (55, −57, 29)	Sub-gyral		0.1/0.4	2.6 (−24, 11, −11)/3.0 (26, 11, −11)
Precuneus	7, 19	0.0/0.4	NS/2.9 (42, −70, 42)				
Cingulate gyrus	31	0.0/0.4	NS/2.5 (4, −39, 41)				
Declive		0.6/0.0	2.4 (−22, −82, −18)/NS				
**COMPONENT 26**				**COMPONENT 23**			
Superior temporal gyrus	22, 38	8.3/4.3	21.1 (−38, 13, −16)/12.7 (42, 11, −12)	Precentral gyrus	3, 4, 6, 13, 43, 44	13.1/16.1	8.1 (−59, −5, 22)/8.2 (61, −1, 18)
Inferior frontal gyrus	13, 47	4.8/4.4	18.3 (−34, 13, −17)/13.9 (40, 15, −14)	Postcentral gyrus	1, 2, 3, 40, 43	5.5/5.7	7.1 (−61, −5, 15)/7.1 (61, −5, 15)
Sub-gyral	13, 21	1.2/0.6	12.8 (−42, 9, −9)/11.4 (44, 11, −9)	Inferior frontal gyrus	9, 44, 45	0.8/2.9	3.3 (−51, 1, 24)/6.0 (61, 5, 24)
Extra–Nuclear	13, 47	0.7/1.1	9.5 (−38, 11, −9)/8.8 (40, 9, −9)	Superior temporal gyrus	22, 38	0.8/1.2	4.9 (−61, −4, 8)/5.6 (61, −2, 7)
Insula	13	1.6/1.3	7.6 (−44, 4, −5)/7.5 (44, 11, −6)	Transverse temporal gyrus	41, 42	0.4/0.5	4.9 (−59, −11, 13)/5.5 (65, −7, 13)
Uncus	28	0.3/0.1	5.4 (−30, 7, −21)/4.5 (28, 9, −19)	Sub-gyral		0.1/0.5	3.1 (−48, −9, 15)/3.8 (50, −7, 17)
Parahippocampal gyrus	28, 34	1.2/0.4	4.6 (−24, 3, −15)/3.8 (22, 5, −15)	Middle frontal gyrus	6, 8, 9	0.0/1.0	NS /3.8 (59, 8, 35)
Subcallosal gyrus	34	0.1/0.3	3.6 (−24, 7, −14)/4.3 (26, 5, −14)	Insula	13	0.9/2.2	2.4 (−38, −9, 15)/3.7 (46, −5, 11)
Middle temporal gyrus	21	0.7/0.0	3.6 (−48, −1, −12)/NS	Extra-Nuclear		0.1/0.2	2.4 (−46, −14, 23)/3.1 (46, −3, 15)
Inferior semi-lunar lobule		0.1/0.1	2.5 (−2, −64, −41)/2.8 (2, −60, −42)	Medial frontal gyrus	6	0.5/1.1	2.5 (0, 2, 48)/3.1 (2, −1, 55)
Third ventricle		0.0/0.1	NS/2.8 (0, −21, 1)	Cingulate gyrus	24	0.1/0.4	2.2 (−2, 4, 44)/2.8 (2, 4, 44)
Thalamus		0.1/0.1	2.2 (−4, −23, 7)/2.7 (4, −21, 5)	Superior frontal gyrus	6	0.1/0.4	2.2 (−4, −3, 65)/2.7 (2, 5, 51)
Anterior cingulate		0.2/0.0	2.6 (0, 23, −10)/NS				
Cerebellar tonsil		0.2/0.1	2.6 (−4, −51, −40)/2.1 (4, −48, −36)				

*NS, Not Survived*.

**Figure 3:**
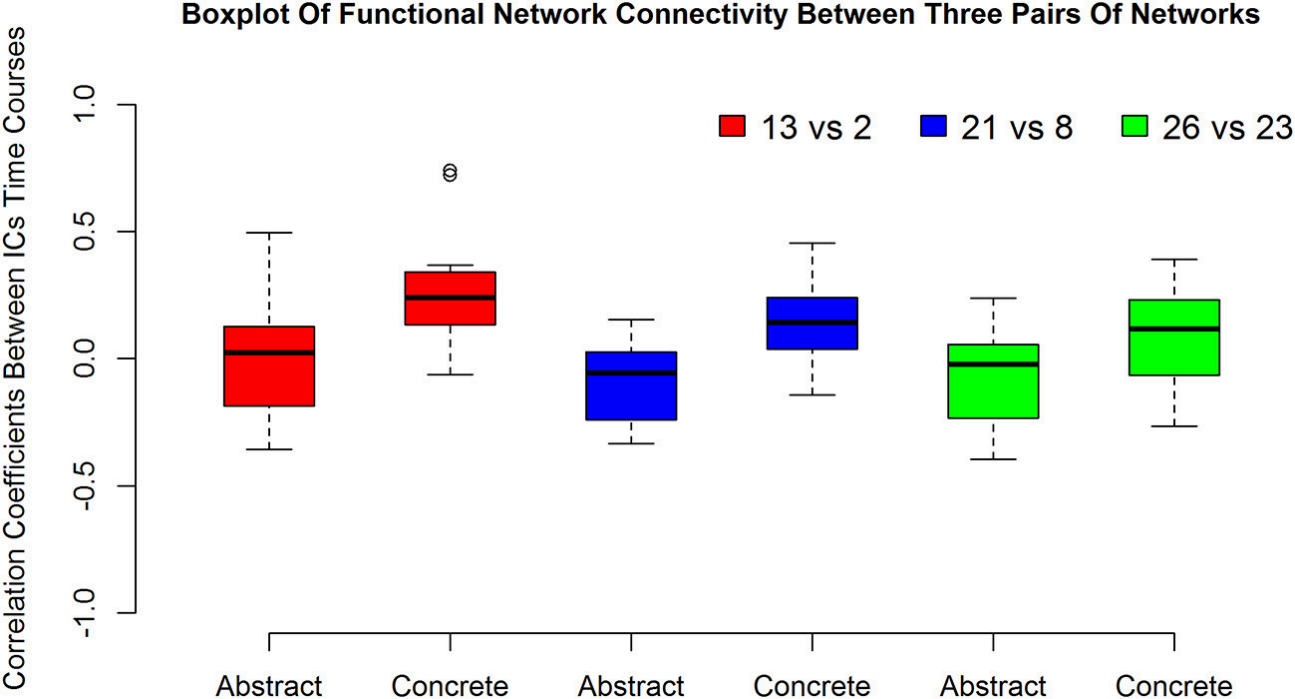
The first, second and the third pairs of boxplots show functional network connectivity between network number 13 vs. 2, 21 vs. 8, and 26 vs. 23, respectively during abstract and concrete imagery for 12 subjects.

The boxplot in Figure 3 suggests how the connections of networks 2 vs. 13 in left, 21 vs. 8 in middle and 26 vs. 23 in the right is altered with the type of imagined characteristics (abstract and concrete).

## Discussion

### Overview

The purpose of this study was to determine how different would be the functional network connectivity during the imagery of abstract characteristics compared to that of concrete characteristics. Previous imaging and behavioral studies which compared the abstract and concrete characteristics had indicated that the concrete words had been processed faster, remembered easier and learned earlier during development. Several neuroimaging studies have also shown there are specific brain regions (particularly in the right hemisphere) which are activated during concrete word processing with higher activation.

Functional network connectivity is a method based on group spatial independent component analysis. This method provides a framework to evaluate the interaction between functional networks. Markedly, this method does not use any anatomical information about positions of voxels to infer the functional networks during abstract and concrete mental imagery. As a result, any consistent structure that has been emerged from the analysis must be driven by the structure in the data and not by prior hypotheses about specific functional organizations of abstract or concrete mental imagery or its anatomical parcellation. In this study, the FNC was performed on fMRI data during abstract and concrete word imagery tasks. Following by that, a two-sided paired *t*-test was used in order to compare the FNC between all possible combinations of 23 networks to address the doubt of whether the connectivity of the functional networks during abstract imagery is significantly different from that of concrete or not.

**Figure 4:**
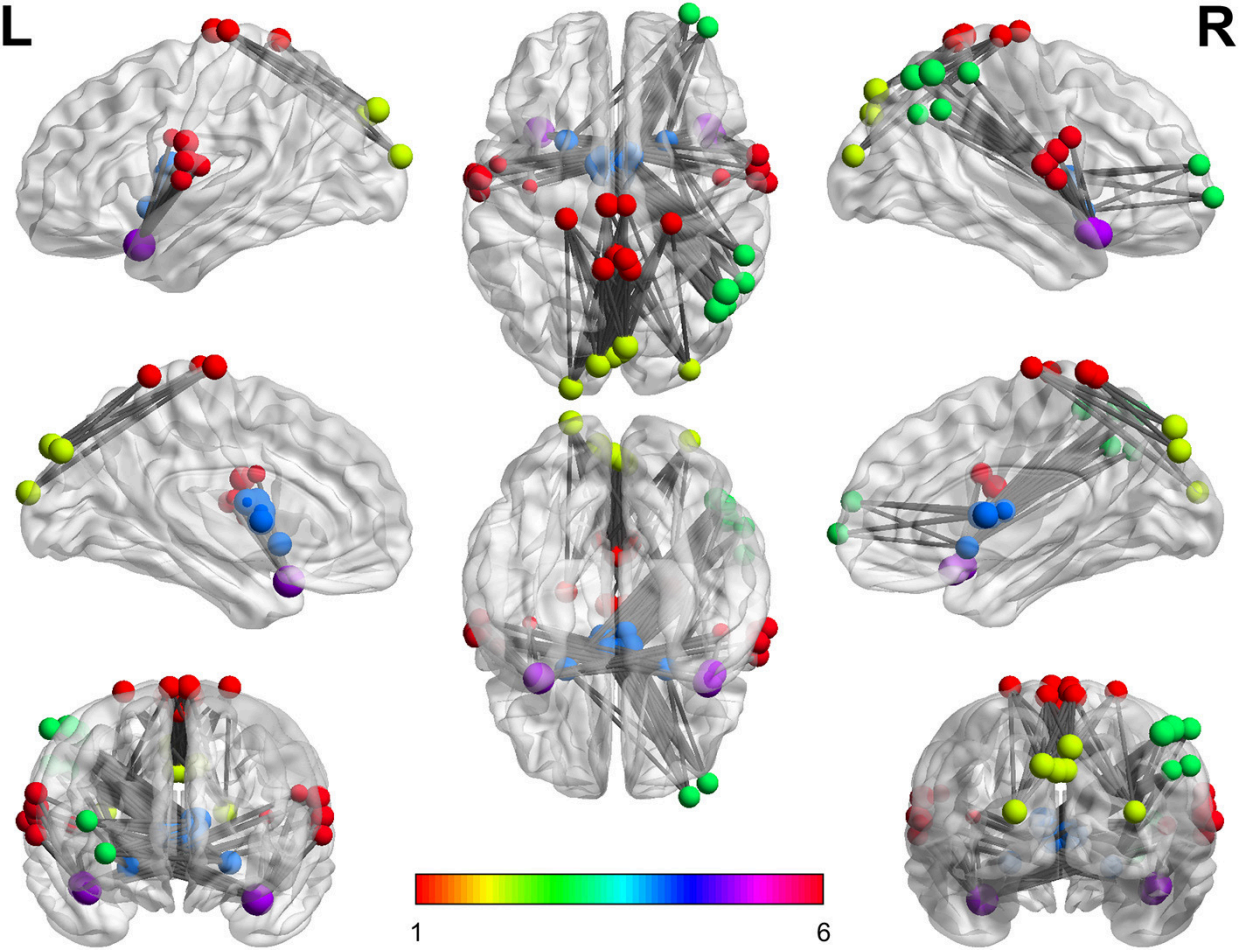
The stronger connections during concrete imagery. Every color is corresponding to a specific network number. As can be seen, there are numerous stronger connections during the concrete imagery than that of abstract and the nodes include both linguistic and visual regions.

Overall, findings show that the connectivity between three pairs of functional networks during abstract words imagery in contrast to concrete words is significantly different. Notably, in all cases, the connectivity among network pairs during concrete imagery is much higher (FDR-adjusted for multiple tests problem, *q* = 0.05). Findings suggest the existence of condition-specific functional connectivity pattern and strength which have been concluded based on whole brain group analysis. Specifically, the results indicate that the brain is more coherent during concrete word imagery and this result may explain the concreteness effect. In other words, stronger connections among functional networks could justify faster processing of concrete words in the brain. Figure 4 Xia et al. (2013) summarizes the results of this paper and indicates stronger connections during concrete imagery compared to abstract between three pairs of networks. In this figure, each color is corresponding to a specific network, and the node sizes are proportional to maximum z-values. It can be seen more coherent networks include both linguistic and visual regions, and clearly the right hemisphere is in higher engagement (include a higher number of more coherent areas) during concrete imagery. The findings propose the results are in agreement with the dual coding theory. Also, as it has been discussed in the next section almost all nodes in this figure are the key regions which have been cited many times in concrete—abstract word processing contrast studies.

### Anatomic Regions of More Coherent Functional Networks and Contrasting Regions

The results implied that the second component contains regions mainly in parietal and frontal lobes such as paracentral lobule, medial frontal gyrus, post-central gyrus, and precentral gyrus. In other side, the 13th component consists of precuneus, cuneus, and middle occipital gyrus. The results revealed that the connectivity between these two components is higher during concrete imagery compared to abstract imagery. Component 13 indicates the substantial role of visual areas at the time of concrete word processing. Exempt for paracentral Lobule, all mentioned regions in this component had reliable activation during mental imagery (Ganis et al., 2004). Middle occipital gyrus and precentral gyrus were two of the five ROIs which their voxels showed significant accuracy in decoding abstract and concrete concepts (Wang et al., 2013). About activation of precentral gyrus, there is inconsistent evidence. In a study by Mellet et al. (1998) precentral gyrus area had a higher activation during concrete condition whereas in the other study by Binder et al. (2005) this region was more responsive to abstract concepts compared to concrete. Direct comparison of concrete and abstract conditions revealed that imagining concrete words partially increases cerebral blood flow in the middle occipital gyrus (Whatmough et al., 2004). Although a greater activation for abstract imagery was found in the precuneus by D'Esposito et al. (1997), there are some studies which reported stronger activation during concrete concepts for precuneus; (Binder et al., 2005; Wang et al., 2010). Additionally, the former studies have shown that the precuneus engaged in the variety of mental imagery and motor tasks. The fact that precuneus is part of a more coherent network during concrete imagery is in line with the dual coding theory which suggests concrete concepts representation is simplified by an extra imagery coding system, because of the more detailed perceptual information relative to the abstract concepts (Wang et al., 2013).

According to the results, the 21st component primarily involves right inferior parietal lobule, right superior frontal gyrus, right middle frontal gyrus and the right supramarginal gyrus, whereas the 8th component covers subcortical areas like extra-nuclear, lateral ventricle, caudate, thalamus, lentiform nucleus, subcallosal gyrus, and parahippocampal gyrus. Figure 3 boxplot proposes stronger functional connectivity between these two networks in concrete imagery in contrast with abstract words evaluation. Network number 21 is the only more coherent network at the time of concrete imagery which exists totally in the right hemisphere. The existence of such network could signify the role of the right hemisphere in concrete word processing and is in agreement with dual coding theory. The relation of inferior parietal lobule with semantic categorization feature integration was shown by Koenig et al. (2005). Moreover, the fact that this region is significantly more harmonized with the other functional network during concrete imagery to abstract, may suggest distinct neural representations on a semantic contrast level which is an aftereffect of the differences in either associate verbal contexts or perceptual-motor information from mental imagery. Based on previous works, inferior parietal lobule, middle frontal gyrus, supramarginal gyrus elicited more activations during concrete imagery than abstract word imagery (Mellet et al., 1998; Sabsevitz et al., 2005). In a study by D'Esposito et al. (1997), it is shown that the right superior frontal gyrus had a stronger activity during abstract mental imagery compared to concrete imagery. In another study, right superior frontal gyrus activated only in the concrete–non-word contrast and not abstract–non-word contrast (Binder et al., 2005). Although these results seem inconsistent, the previous finding suggests this area plays a central role in abstract-concrete imagery contrasts. Ganis et al. (2004) showed this region has solid activation during mental imagery. Ganis et al. (2004) also showed areas including caudate, thalamus, lentiform nucleus, and parahippocampal gyrus are activated during imagery and perception and Sabsevitz et al. (2005) reported a positive correlation between task difficulty also amount of activation in caudate, thalamus, the lentiform nucleus in the semantic similarity judgment task. Sabsevitz et al. (2005) also found stronger activation for concrete nouns in the subcallosal gyrus and parahippocampal gyrus in contrast to abstract nouns.

Two main areas which play a pivotal role in functional network number 26 and significantly forming this component are superior temporal gyrus with stronger activation and bigger volume in the left hemisphere and inferior frontal gyrus. There are some reports which propose the abstract concepts elicit greater activity in both regions in contrast to concrete concepts (Wise et al., 2000; Wang et al., 2010) and surprisingly these two have been identified by group ICA as a network. In other words, they have a similar pattern of activation during performing this task. The superior temporal gyrus has been related to the effect of semantic context (Van Petten and Luka, 2006) and semantic judgment task (D'Esposito et al., 1995). As the performed task in this study is based on the judgment of animal concrete and abstract characteristics, extraction of this region as the functional network seems reasonable. The second dominant area within component 26 is inferior frontal gyrus which a study based on semantic decision task by Goldberg et al. (2007) showed that this area is sensitive to the concept abstractness. Component 23 mainly incorporated anatomic areas including the precentral gyrus, post-central gyrus, and partially inferior frontal gyrus, superior temporal gyrus, transverse temporal gyrus, and insula. Both insula and transverse temporal gyrus are engaged in mental imagery task (Ganis et al., 2004). Moreover, significant activation and deactivation for both concrete and abstract stimuli vs. baseline were shown in these two areas (Kiehl et al., 1999). According to Figure 3 and these explanations, one may conclude that the connection between superior temporal gyrus, inferior frontal gyrus in one IC and the precentral gyrus, post-central gyrus and partially, transverse temporal gyrus and insula in another IC is significantly higher during concrete imagery.

In order to compare the brain connectivity between concrete and abstract words, other psycholinguistic parameters are needed to be matched for two blocks in the paradigm. The concrete and abstract words were matched for number of syllables (concrete word: mean, 2.8125 and *SD* 0.7803; abstract words: mean, 2.7188 and *SD* 0.7719; *p* = 0.6307). The statistics of word length (number of letters) for abstract and concrete words are (concrete word: mean, 6.3750 and *SD* 1.2115; abstract words: mean, 5.6563 and *SD* 1.4053; *p* = 0.0322).Thus, there was a small difference between the average word length of abstract and concrete words (*p* = 0.0322). However, it is noteworthy that since the stimuli are presented verbally (by audio) in the native language, number of syllables is more effective (in pronunciation and voice length) than the number of letters (which may be more considerable in written expressions), and number of the syllabus are matched in this study. Thus, the marginal difference in the average number of letters is unlikely to induce effect in brain response. Besides, the frequency of abstract and concrete words has not been compared in a quantitative manner. Nevertheless, the effect of these issues may be examined in the future studies with exactly matched stimuli.

### Validation

To validate the results using an extra statistical baseline, the block of concrete word imagery data is divided into two parts (i.e., concrete 1, concrete 2) and the connectivity among all possible pairs is compared. After FDR correction, no pair is found with significant different connectivity. More precisely, all *p-*values were higher than 0.01. Furthermore, abstract word imagery is also divided data into two parts (i.e., abstract 1, abstract 2) and connectivity among all possible pairs is compared as well. After FDR correction, no pair with significantly different connectivity survived.

To validate this claim that the brain is more coherent at the time of concrete imagery, functional network connectivity distribution (FNCD) is evaluated. In the current dataset, there are $\frac{23\;\times \;\left(23-1\right)}{2}\times 12=3,036\;$correlation coefficients between network pairs for all subjects. The distribution of these correlation coefficients for the abstract and concrete imagery have been compared using the Wilcoxon rank-sum test. This statistical test was used to determine if two distributions are significantly different. The resulted *p*≅0 indicated that the distributions of functional network connectivity which have been extracted from all subjects are different for the abstract and concrete imagery. One tail paired *t*-test at the 0.05 significance level was performed to see whether mean of whole brain integration (correlation coefficients between all functional network time courses for all subject) is higher during concrete imagery compared to abstract or not. Since the sample size of vectors (containing all correlations) was relatively big; i.e., 3,036, even a small difference between the mean of the vectors may be significant. This is called practical significant problem, and that is the difference is statistically significant but practically. In order to avoid this problem, a random sample with a size of 500 from each vector was taken, and the hypothesis testing was performed on two resulted sample vectors. One tail paired *t*-test led to 3.5180 × 10^−4^ *p*−*value*, so the null hypothesis is rejected and mean whole brain integration is higher at the time of concrete imagery. The paired *t*-test on 1, 000 different random samples of FNCD is repeated to check whether the result of the hypothesis testing is biased by the sampling randomness or not. The result shows the *p*-value is only 5 times higher than 0.05. So, the results are robust and mean of FNCD is higher over concrete word imagery.

**Figure 5:**
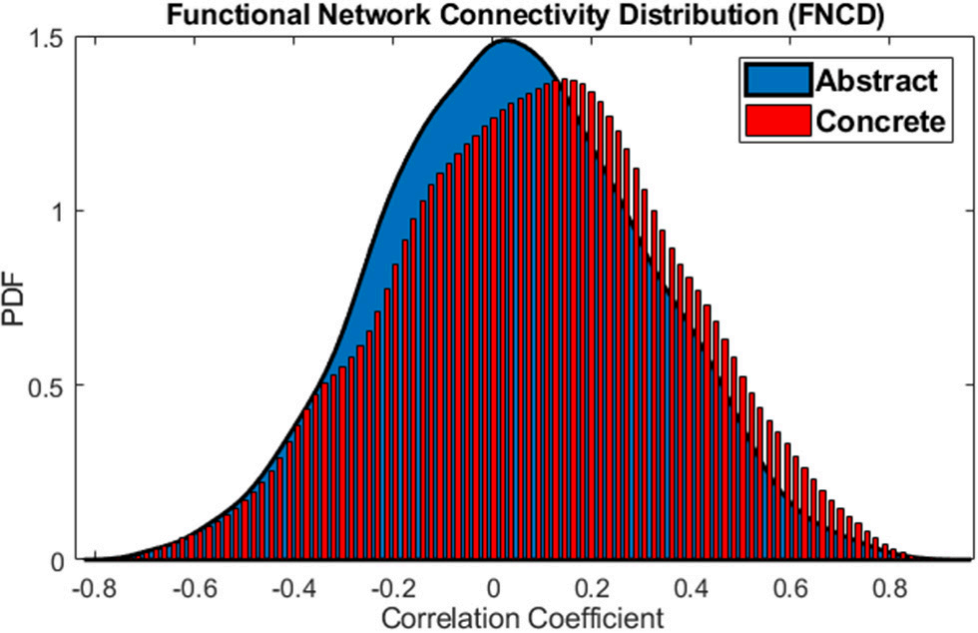
Functional Network Connectivity Distributions for the abstract (blue) and concrete word (red) imagery. The brain is in the more coherent state over concrete imagery.

The functional network distributions of abstract and concrete words imagery have been shown in Figure 5. This Figure apparently shows how the brain is more integrated (FNCD include stronger correlations) all along concrete imagery.

**Figure 6:**
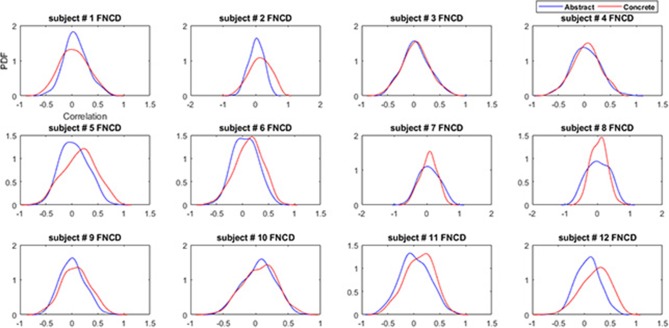
Functional network connectivity distributions for the abstract (blue) and concrete word (red) imagery across subjects.

The distribution of functional network correlations the single subject level are reported in Figure 6. As it can be seen the concrete FNCDs tend to be more left-skewed (include higher connectivity) almost for all subjects compared to abstract FNCD.

To study the skewness of concrete and abstract FNCD quantitatively and more accurate, the skewness of abstract and concrete FNCD for every subject was calculated using sample skewness which defined as:

(1) $$\begin{array}{l} Sample-skewness=\frac{\sqrt{n\left(n-1\right)}}{n-2}\frac{m_{3}}{m_{2}^{3/2}} \end{array}$$

Where *n* is the sample size, *m*_3_ the third moment and *m*_3_ is sample variance. In this way, two vectors (one for abstract and one for concrete) with the size of 12 × 1 for all subjects were calculated. In order to evaluate whether the concrete FNCDs are significantly left-skewed (have negative values for skewness) compared to abstract, the one tail paired *t*-test at the 0.05 significance level was applied (3.4972 × 10^−6^ *p*−*value*). This result suggests concrete FNCDs across subjects are significantly more left-skewed or equally more likely to include higher correlations in contrast to abstract FNCD.

**Figure 7:**
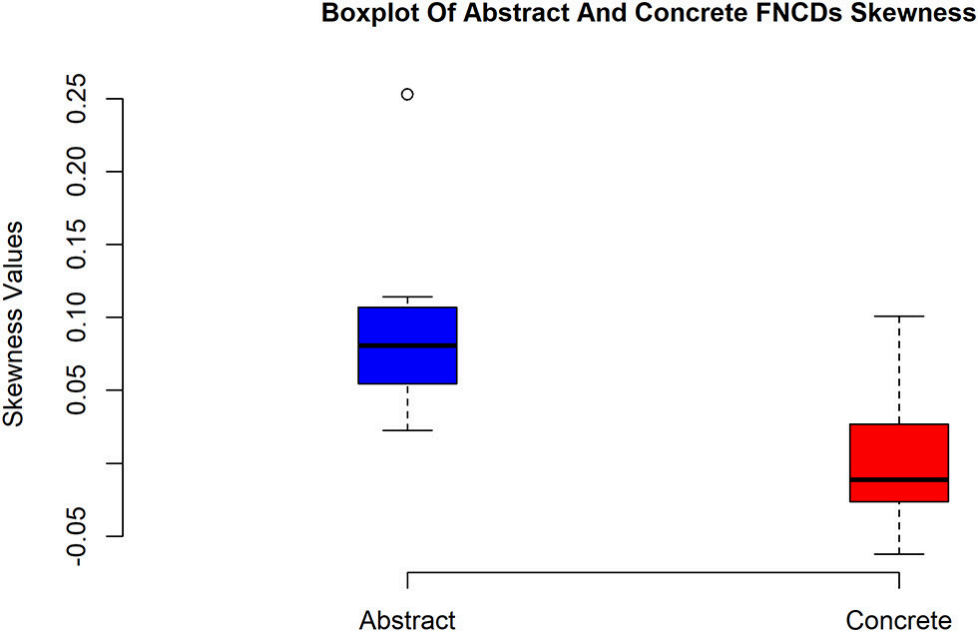
Box plots of skewness values of abstract and concrete FNCDs. As can be seen, the concrete FNCDs are more left-skewed than abstract FNDCs.

The boxplot in Figure 7 illustrates how concrete FNCDs across subjects are significantly more left-skewed or equally the brain is in the more coherent state over concrete imagery in contrast to abstract.

**Figure 8:**
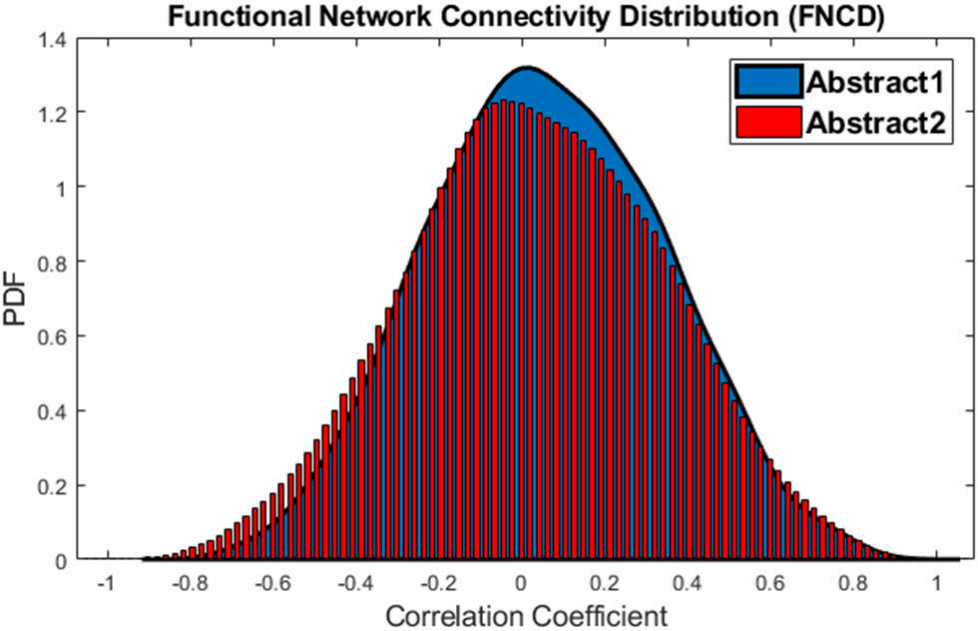
The distributions of Functional Network Connectivity values for the abstract 1 (blue) and abstract 2 (red).

Also, a statistical baseline is provided for the whole brain integration analysis. To this aim, connectivity between all networks is calculated for each of abstract 1 and abstract 2 sub-groups which are two vectors with the size of 3,036. Again, in order to avoid practical significance problem, the paired *t*-test is applied 1,000 times on 1,000 different sample vectors with a size of 500. Result revealed that in 44% of times *p* < 0.05 (or equally in 44% of times mean of the vectors are different). This implies that the difference between average functional network connectivity during abstract 1 and abstract 2 is not significant. The distributions of functional network connectivity for abstract 1 and abstract 2 sub-groups have been shown in Figure 8.

**Figure 9:**
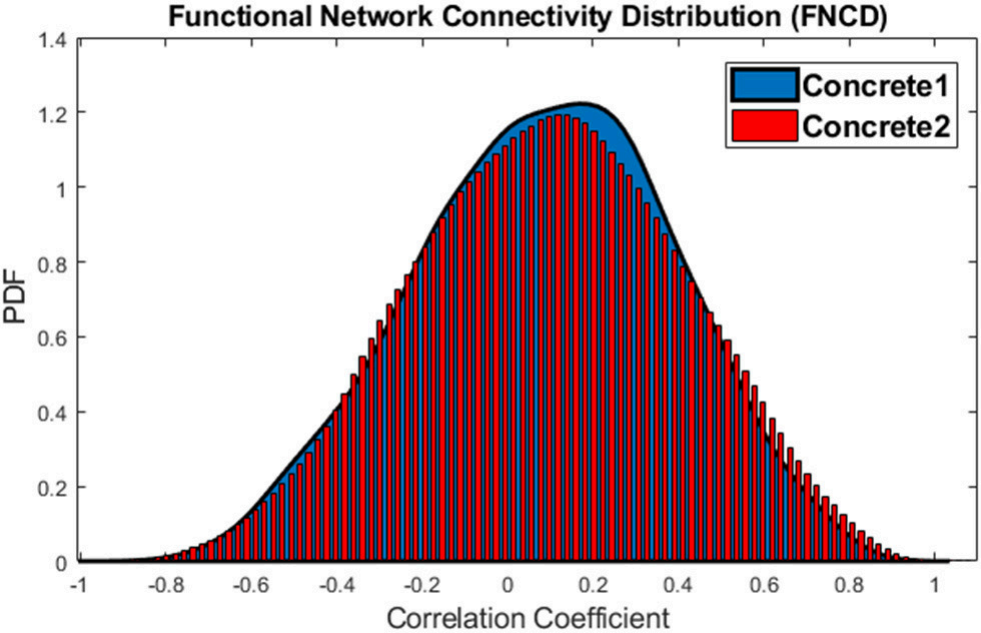
The distributions of Functional Network Connectivity values for the concrete 1 (blue) and concrete 2 (red).

This procedure also is performed for the concrete 1 and concrete 2 sub-groups, and just in 9% of times, the *p* < 0.05 which means that the difference is not significant. The distributions of functional network connectivity for concrete 1 and concrete 2 words imagery have been shown in Figure 9.

As can be seen from the results, the functional connectivity of brain networks is generally higher for imagery of concrete vs. abstract properties. This is consistent with other research which compared the brain activity between abstract and concrete imagery. These papers permutated the order of abstract and concrete blocks (Binder et al., 2005; Papagno et al., 2013). Since the obtained results on connectivity are consistent with increase in activity, thus one may conclude that the observed effect is notz due to the fatigue or order of presenting the concrete and abstract features. Moreover, for each subject, the abstract trials take just 3.5 min, so the practice or fatigue effects are not acute.

### Future Works

In this study, a comparison for functional connectivity is performed during abstract and concrete imagery using a semantic decision task with auditory stimuli. Some other investigations used visual stimuli to study concreteness effect. So, investigating the effect of the type of stimuli (auditory or visual) would be a possible research direction. There are various methods including clustering algorithms and seed based methods available in order to study functional connectivity. In future studies, graph theoretical measures can be used to quantify the global pattern of connectivity strength and synchronization among brain networks and compare them between abstract and concrete imagery.

## Author Contributions

All authors listed have made a substantial, direct and intellectual contribution to the work, and approved it for publication.

### Conflict of Interest Statement

The authors declare that the research was conducted in the absence of any commercial or financial relationships that could be construed as a potential conflict of interest. The reviewer AA and handling Editor declared their shared affiliation at the time of review.
