# Supplementary material for: Distinct Functional Network Connectivity for Abstract and Concrete Mental Imagery
Source: Front Hum Neurosci. 2018 Dec 18;12:515. doi: 10.3389/fnhum.2018.00515 (PMC6305479; doi:10.3389/fnhum.2018.00515)
Supplement: Supplementary file 1 [file Table_1.DOCX]

Supplementary Material

Distinct functional network connectivity for abstract and concrete mental imagery

Sobhan Hemati^1^, Gholam-Ali Hossein-Zadeh ^1*^

**Correspondence:**

Gholam-Ali Hossein-Zadeh: [ghzadeh@ut.ac.ir](mailto:ghzadeh@ut.ac.ir)

In order to assess the match of some of the psycholinguistic variables, the empirical assessment was performed by twenty students. They were asked to rate the concreteness of the animal characteristics (used in the paradigm) based on “whether the characteristic relies more on directly perceived experiences and it is more imageable or indirectly learned verbal knowledge and it is less imageable.” The abstract-concrete scale range was from 1 to 7, in which 1 indicated highly abstract (difficult to image) and 7 indicated highly concrete (easy to image). We defined an abstract characteristic for the participants as one that would be typically learned indirectly through verbal facts and cannot be experienced through their sense or action, whereas a concrete characteristic was explained as one that could be directly perceived through one of the five senses. The average of concreteness measure for concrete words used in our paradigm (mean, 6.089 and SD 1.36) was significantly higher$(p-value\cong0$) than that of abstract words (mean, 3.094 and SD 1.978). The concrete and abstract words were matched for psycholinguistic variables including number of syllables and word length. These are summarized in Table 1.

Table 1: mean and standard deviation values for concreteness, number of syllables and word length of abstract and concrete words.

|  | Concreteness  (mean, SD) | Number of syllables (mean, SD) | Word length  (mean, SD) |
| --- | --- | --- | --- |
| Abstract words | 2.8125, 1.7427 | 2.7188, 0.7719 | 5.6563, 1.4053 |
| Concrete words | 6.0469, 1.3014 | 2.8125, 0.7803 | 6.3750, 1.2115 |

Table 2 provides complete information about the abstract and concrete words used in our study. It is noteworthy that the length and syllabus information refer to Persian words used in the study, and the English translation is provided for the sake of completeness.

Table 2: number of syllables, word length for every abstract and concrete word. Average and standard deviation concreteness values, across subjects. A=Abstract; C=Concrete

| Mean of concreteness across subjects | Standard Deviation of concreteness across subjects | Our Classification | English | Length | Syllables | Persian |
| --- | --- | --- | --- | --- | --- | --- |
| 6.1000 | 1.5951 | C | Has crawl | 9 | 5 | دارای پنجه |
| 1.8000 | 1.8738 | A | Nocturnal | 4 | 2 | شب زی |
| 6.3000 | 1.6364 | C | Long neck | 8 | 4 | گردن بلند |
| 2.7000 | 1.5670 | A | Crafty | 7 | 3 | فریب کار |
| 2.1000 | 1.8529 | A | Aquatic | 4 | 2 | آب زی |
| 6.3000 | 1.5670 | C | Narrow tail | 7 | 3 | دم باریک |
| 3.0000 | 1.8856 | A | Timid | 4 | 2 | ترسو |
| 6.4000 | 1.5776 | C | Long ears | 7 | 3 | گوش دراز |
| 2.1000 | 1.4491 | A | Clever | 7 | 4 | هوش بالا |
| 3.5000 | 1.9003 | A | Fierce | 5 | 3 | درنده |
| 6.0000 | 1.8856 | C | Leggy | 7 | 4 | پای بلند |
| 6.5000 | 0.8498 | C | Maned | 6 | 2 | یال دار |
| 3.9000 | 2.2828 | A | Benign | 6 | 3 | بی آزار |
| 5.9000 | 1.9692 | C | Sharp claw | 7 | 3 | تیز پنجه |
| 2.4000 | 1.4298 | A | Shameless | 5 | 2 | بی شرم |
| 5.8000 | 1.9322 | C | Sharp teeth | 8 | 3 | تیز دندان |
| 1.9000 | 1.5239 | A | Loyal | 5 | 3 | باوفا |
| 6.1000 | 1.1005 | C | Tiny body | 7 | 4 | جثه کوچک |
| 2.0000 | 1.4142 | A | Noble | 4 | 2 | نجیب |
| 1.7000 | 1.5670 | A | Herbivorous | 8 | 3 | گیاه خوار |
| 6.1000 | 1.5239 | C | Hoofed | 5 | 2 | سم دار |
| 2.9000 | 2.0248 | A | Dull | 8 | 4 | هوش پایین |
| 5.9000 | 1.5239 | C | Long muzzle | 8 | 4 | پوزه دراز |
| 3.2000 | 1.7512 | A | Cacophonous | 5 | 3 | بد صدا |
| 2.7000 | 1.8886 | A | Gourmand | 5 | 2 | پر خور |
| 3.4000 | 1.7764 | A | Dirty | 4 | 2 | کثیف |
| 5.7000 | 1.4181 | C | Horned | 6 | 2 | شاخ دار |
| 2.7000 | 1.4181 | A | Playful | 7 | 3 | بازیگوش |
| 5.8000 | 1.9322 | C | Straight neck | 7 | 3 | گردن صاف |
| 5.5000 | 1.6499 | C | Big wings | 7 | 3 | بزرگ بال |
| 3.5000 | 1.5092 | A | Carnivorous | 8 | 2 | گوشتخوار |
| 6.0000 | 1.4907 | C | Straight legs | 5 | 2 | صاف پا |
| 3.7000 | 1.6364 | A | Reptile | 5 | 3 | خزنده |
| 3.3000 | 2.1108 | A | Oviparous | 7 | 3 | تخم گذار |
| 6.4000 | 0.5164 | C | Beast | 6 | 2 | چهار پا |
| 5.8000 | 1.0328 | C | Feathery | 6 | 3 | پر از پر |
| 3.3000 | 1.8288 | A | Euphonic | 6 | 3 | خوش صدا |
| 2.5000 | 1.8409 | A | informant | 6 | 3 | خبرچین |
| 6.5000 | 0.7071 | C | venomous | 6 | 2 | نیش دار |
| 6.2000 | 1.1353 | C | Without tail | 4 | 2 | بی دم |
| 5.6000 | 1.5776 | C | Two feet | 4 | 2 | دو پا |
| 4.6000 | 1.2649 | A | Hard-working | 6 | 2 | سخت کوش |
| 5.7000 | 0.8233 | C | Without antler | 6 | 3 | بی شاخک |
| 3.5000 | 1.1785 | A | Inoffensive | 8 | 5 | بدون آزار |
| 3.1000 | 2.0790 | A | Low apetite | 7 | 3 | کم خوراک |
| 6.1000 | 0.9944 | C | Soft skin | 6 | 2 | نرم پوست |
| 6.0000 | 1.3333 | C | Wide head | 5 | 2 | سر پهن |
| 2.5000 | 1.4337 | A | Clean | 4 | 2 | تمیز |
| 6.1000 | 1.2867 | C | Has comb | 6 | 2 | تاج دار |
| 3.5000 | 2.1213 | A | Speaker | 5 | 3 | سخنگو |
| 5.9000 | 0.9944 | C | Thick-tailed | 6 | 3 | دم کلفت |
| 6.4000 | 0.8433 | C | Bent beak | 7 | 3 | منقار خم |
| 2.9000 | 1.2867 | A | Domestic | 4 | 2 | اهلی |
| 5.8000 | 1.3984 | C | Wooly | 6 | 3 | پشمالو |
| 6.0000 | 1.1547 | C | Short tail | 7 | 3 | دم کوتاه |
| 3.4000 | 2.0111 | A | Colorful | 7 | 3 | رنگارنگ |
| 6.0000 | 0.8165 | C | Small wings | 7 | 3 | کوچک بال |
| 6.2000 | 0.6325 | C | hairy | 4 | 2 | پر مو |
| 6.2000 | 0.9189 | C | Long tail | 6 | 3 | دم دراز |
| 1.9000 | 0.9944 | A | Shy | 6 | 4 | خجالتی |
| 1.6000 | 0.6992 | A | impish | 4 | 2 | موذی |
| 2.2000 | 1.1353 | A | Lazy | 4 | 2 | تنبل |
| 6.2000 | 1.2293 | C | Sharp beak | 8 | 3 | منقار تیز |
| 2.5000 | 1.5092 | A | sharp-sighted | 6 | 2 | تیز بین |
